# African Pygmies

**Published:** 1891-08

**Authors:** 


### AFRICAN PYGMIES.

Stanley in his London lecture before the Geographical Society, says: "Over a country half as large again as France, covered with huge trees--ten thousand millions of them, calculates the traveller himself—standing so thick that it is always twilight below their interwoven branches, wander in thousands, and have wandered for three thousand years at least, a race of light-brown men and women—the color of half-baked bricks—scarcely four feet high.

In the course of centuries, they have so fitted themselves to their environment, that the dreary forest, where full light never falls, and every shadow across the sun produces a kind of penetrable night, has became to them their world. They know of nothing beyond it, even by tradition, have no idea of the great prairies outside, have never seen grass growing in quantities, cannot to all appearance conceive of a country in which trees are not, or in which movement does not involve a painful threading through the bush. The only spaces they know are the small oases, where larger natives have made clearances in which to plant gardens of the banana, which in this country reaches maturity in twelve months, and serves all the purposes of the cereals in more favored lands. The little people, taught by ages of experience, know their forests thoroughly, can tell exactly what is edible and what poisonous, and can find food everywhere ; but the bananas draw them irresistibly from the lonelier depths. They plant their villages around the oases in order to get the fruit, sometimes paying the cultivators by their services as trackers and watchmen, which their superior knowledge of the woods enables them to offer, but more frequently feeding without leave on crops practically inexhaustible.

These little people have become adapted to their surroundings, and would doubtless perish, with other arboreal creatures, if the forests were destroyed. The discovery of a new race of men is certainly an event of the highest interest, and if these little savages be not the missing link between man and the simians, we may expect them to supply much desired knowledge concerning primitive conditions. Both from revelation and science we learn that primitive man was an arborean. Now we shall know what an arborean, pure and simple, is.

